# Influence of Population Density for COVID-19 Spread in Malaysia: An Ecological Study

**DOI:** 10.3390/ijerph18189866

**Published:** 2021-09-18

**Authors:** Kurubaran Ganasegeran, Mohd Fadzly Amar Jamil, Alan Swee Hock Ch’ng, Irene Looi, Kalaiarasu M. Peariasamy

**Affiliations:** 1 Clinical Research Center, Seberang Jaya Hospital, Ministry of Health Malaysia, Seberang Perai 13700, Penang, Malaysia; fadzly.crc@gmail.com (M.F.A.J.); alanchng1978@gmail.com (A.S.H.C.); irenelooi@yahoo.com (I.L.); 2 Medical Department, Seberang Jaya Hospital, Seberang Perai 13700, Penang, Malaysia; 3 Institute for Clinical Research, National Institutes of Health, Ministry of Health Malaysia, Setia Alam 40170, Selangor, Malaysia; drkalai@moh.gov.my

**Keywords:** population density, clusters, urbanization, COVID-19, Malaysia

## Abstract

The rapid transmission of highly contagious infectious diseases within communities can yield potential hotspots or clusters across geographies. For COVID-19, the impact of population density on transmission models demonstrates mixed findings. This study aims to determine the correlations between population density, clusters, and COVID-19 incidence across districts and regions in Malaysia. This countrywide ecological study was conducted between 22 January 2021 and 4 February 2021 involving 51,476 active COVID-19 cases during Malaysia’s third wave of the pandemic, prior to the reimplementation of lockdowns. Population data from multiple sources was aggregated and spatial analytics were performed to visualize distributional choropleths of COVID-19 cases in relation to population density. Hierarchical cluster analysis was used to synthesize dendrograms to demarcate potential clusters against population density. Region-wise correlations and simple linear regression models were deduced to observe the strength of the correlations and the propagation effects of COVID-19 infections relative to population density. Distributional heats in choropleths and cluster analysis showed that districts with a high number of inhabitants and a high population density had a greater number of cases in proportion to the population in that area. The Central region had the strongest correlation between COVID-19 cases and population density (*r* = 0.912; 95% CI 0.911, 0.913; *p* < 0.001). The propagation effect and the spread of disease was greater in urbanized districts or cities. Population density is an important factor for the spread of COVID-19 in Malaysia.

## 1. Introduction

Humans are known to spread infectious diseases, such as the ancient bubonic plague, the human immunodeficiency virus (HIV), and the current ongoing coronavirus disease (COVID-19) [1,2]. Although global public health mitigation and suppression strategies are being rigorously executed, emerging pathogens with great potency have been successively invading human populations worldwide. Microbial experts and immunologists are consistently postulating heterogenous plausible theories, including genomic expression, cell structure, and the biological environment of microorganisms that are capable of altering the host’s immune responses, thus making people vulnerable to infections [3,4,5]. However, these systemic theories at the molecular and microscopic levels do not answer the fundamental question as to why geometric progressions or the chaotic spread of diseases are sustained at the population level. Environmental attributes within the triad of infectious disease dynamics, apart from host and agent, are crucial factors when scrutinizing risks for pathogen spread at the population level.

With the continual growth of populations, human mobility and interactions become extensively widespread, putting forward an eco-epidemiological assumption that disease transmission at the population level could be caused by the mixing patterns of individuals within the environment. These preliminary assumptions are highlighted within the network models of modern epidemiology [6,7,8,9,10]. Coherent with mixing behaviors, humanity has encountered massive migrations alongside transformations in social, economic, infrastructural, and architectural life changes within societies that are capable of mapping new landscapes of dense populations across territories [11,12,13].

Reflecting on the advancement of medical treatment, diagnostics, testing capacities, and contact tracing approaches, the current COVID-19 pandemic has posed tremendous challenges to epidemiologists in the quest to contain the outbreak. This phenomenon has advanced, a priori, that the proximity between people could be a factor in the spread of infections. While Malaysia nearly flattened the curve during the first and second waves of the pandemic [14], an “unexpected exponential surge” of cases led to the third wave of the pandemic in late 2020 and early 2021. In addition, the absence of a vaccination rollout during this stage of the pandemic could have contributed to the rapid rise of daily cases. Malaysia fundamentally relied on targeted interventions of nontherapeutic measures to equilibrize lives and livelihood, while implementing full or partial movement control orders (MCOs); risk area-based lockdowns; rigorous contact tracing; the closure of social, tourism, education and certain economic sectors; and the restriction of people’s movement [14]. Despite these interventions, Malaysia showed different infection rates and propagation effects. The nation observed a district-wise regional estimate spike of cases that were mainly concentrated in the Central region of Malaysia compared to other regions. Such geographically different intensities of infection were observed in Italy [15], and it was postulated to be attributed to differences in demographics, socio-economic factors, health resource capacities, or political management [15]. Crucially, infections that spread between different geographies were hypothesized to be caused by the magnitude of population density in each area and the size of the area or district.

The impacts of dense populations within different geographical areas on COVID-19 incidence rates are emerging within the literature. While most preliminary findings highlight positive correlations between COVID-19 incidence and the compactness of people [11,13,16,17,18,19], some have yet to reach a consensus [12,20]. These studies measured the direct effect of population density on the incidence count of cases, but did not establish an in-depth analysis that visualized heat distribution and the correlation of cases that could be attributed to attack rates and infectivity per km^2^, which could provide how much risk individuals were exposed to regarding COVID-19 infections in a given area when contacts within compactness were established. Malaysia is currently facing the third phase of the COVID-19 pandemic. To be coherent with the emerging epidemiological landscape, this study aims to determine the correlations between population density, clusters, and COVID-19 incidence across districts and regions in Malaysia. In addition, this study aims to provide an estimated risk of infection attributable to people when a unit of density is increased in a particular district.

## 2. Materials and Methods

### 2.1. Study Population, Design, and Setting 

**Figure 1:**
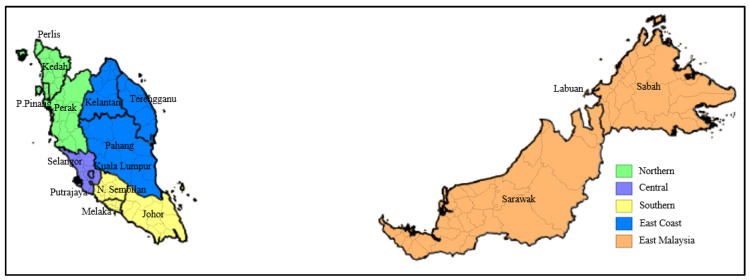
Baseline map of Malaysia. Dark boundaries depict states; light boundaries depict districts within states; color-shaded areas depict regions in Malaysia.

This countrywide ecological study was conducted between 22 January 2021 and 4 February 2021 through spatial epidemiological analytics involving 51,476 reported, active COVID-19 cases across 144 districts within five regions (Northern, Central, East Coast, Southern and East Malaysia) in Malaysia. The study utilized population-level aggregated data. Figure 1 depicts a baseline map of bordering districts, states, and regions in Malaysia.

### 2.2. Data Source

Secondary data was retrieved from multiple sources: the primary data of district-wise 14 day moving data of reported COVID-19 active cases prior to reimplementation of the national-level movement control order (MCO) during Malaysia’s third wave of the pandemic was obtained from the Ministry of Health Malaysia live COVID-19 webpage (https://covid-19.moh.gov.my/ accessed on 4 February 2021); retrieval point—4 February 2021) [21]; the total revised population projection based on countrywide aggregate census data, stratified according to regions and districts for the year 2019, was obtained from the Population and Housing Census of Malaysia, Department of Statistics Malaysia, 2021 [22]; data on the total area (km^2^) was obtained from the Department of Survey and Mapping Malaysia, 2019 [23]; administrative shapefiles and district coordinates were retrieved from the 2019 Malaysia-Subnational Administrative Districts Data, United Nations Office for Coordination of Humanitarian Affairs, 2019 [24]. The retrieved data (available in raw form in Supplementary File Table S1) was subsequently tabulated in a spreadsheet to build a wide geospatial dataset with district coordinates as the unit of analyses. The dataset was converted to a .csv file and imported to the relevant statistical software for further analyses. States within the regional borders of Malaysia were classified according to the National Population Housing Scheme (PRIMA) [25]: the Northern region included Perlis, Kedah, Pulau Pinang, and Perak; the Central region included Selangor, Wilayah Persekutuan Kuala Lumpur, and Wilayah Persekutuan Putrajaya; the Southern region included Negeri Sembilan, Melaka, and Johor; the East Coast region included Pahang, Terengganu, and Kelantan; and East Malaysia included Sabah, Sarawak, and Wilayah Persekutuan Labuan. 

### 2.3. Operators

Population density was calculated as the number of inhabitants living in an area per kilometer square (inhabitants/km^2^) for each district. The number of infected people per km^2^ was computed as the number of reported active cases per square kilometer (number of reported active cases/km^2^) for each district. Attack rate was calculated as the number of reported active cases divided by the total number of people (number of reported active cases/total number of people) for each district [26]. Hotspots were identified as districts with more than 40 reported active cases [21].

### 2.4. Statistical Analysis

Choropleth maps were built to visualize the distributional heat of COVID-19 counts, the number of infected people per km^2^, and attack rates in relation to the projected total number of inhabitants in Malaysia (*n* = 33,531,200). Hierarchical cluster analysis using an average linkage method with a squared Euclidean distance was employed to synthesize a precise dendrogram for classifying districts vulnerable to COVID-19 infections based on population density. The execution of hierarchical cluster analysis aimed to create clusters of districts according to the rate of reported COVID-19 infections and population density, facilitating comparisons between populations of the highest and lowest rates of infection. The synthesized clusters were beyond regional estimates, but conceptually within different geographies for real-life interpretations, postulating that dense areas of infections would probably be distributed across urbanized or rural areas and within cities or metropolitan areas.

The effect size, Pearson’s correlation coefficients (*r*) with their corresponding 95% confidence intervals (CIs), was yielded to determine region-wise correlations between active COVID-19 cases and population density. The *r* value ranges between −1 and +1 [27,28]. A correlation directing towards −1 indicates that two variables are more negatively linearly related, a correlation of 0 means that two variables do not have any linear relations, while a correlation coefficient of +1 means that two variables are directed towards a more perfectly positive linear relation [28,29]. The magnitude size of the *r* effect can be interpreted as follows [30]:

  i. 0.90 to 1.00 (or −0.90 to −1.00) as very high positive (or negative) correlation;
 ii. 0.70 to 0.90 (or −0.70 to −0.90) as high positive (or negative) correlation;
iii. 0.50 to 0.70 (or −0.50 to −0.70) as moderate positive (or negative) correlation;
iv. 0.30 to 0.50 (or −0.30 to −0.50) as low positive (or negative) correlation;
 v. 0.00 to 0.30 (or 0.00 to −0.30) as negligible correlation.

The coefficient of determination (*R*^2^) values were synthesized to determine the strengths of association between cases and population density according to geographical regions in Malaysia. Simple linear models using the equation *Y = B + aX* (*Y* = dependent variable representing COVID-19 cases; *X* = independent variable representing population density; *B* = constant) were deduced at countrywide and regional levels to confirm the associated impact of population density on COVID-19 infections [13,31]. Significant levels were set at two tails (*p* < 0.05). Analysis was conducted using GeoDa version 1.18 (Center for Spatial Data Science University of Chicago, Illinois, IL, USA) and SPSS version 22.0 software (IBM Corp, New York, NY, USA).

### 2.5. Conference Presentation

Findings from this study were presented at the 22nd National Public Health Colloquium, 4–6 May 2021, Kuala Lumpur, Malaysia. 

## 3. Results

### 3.1. Geographic Disparities of COVID-19 Case Counts, Attack Rates, and Infected People Per km^2^ in Malaysia

**Figure 2:**
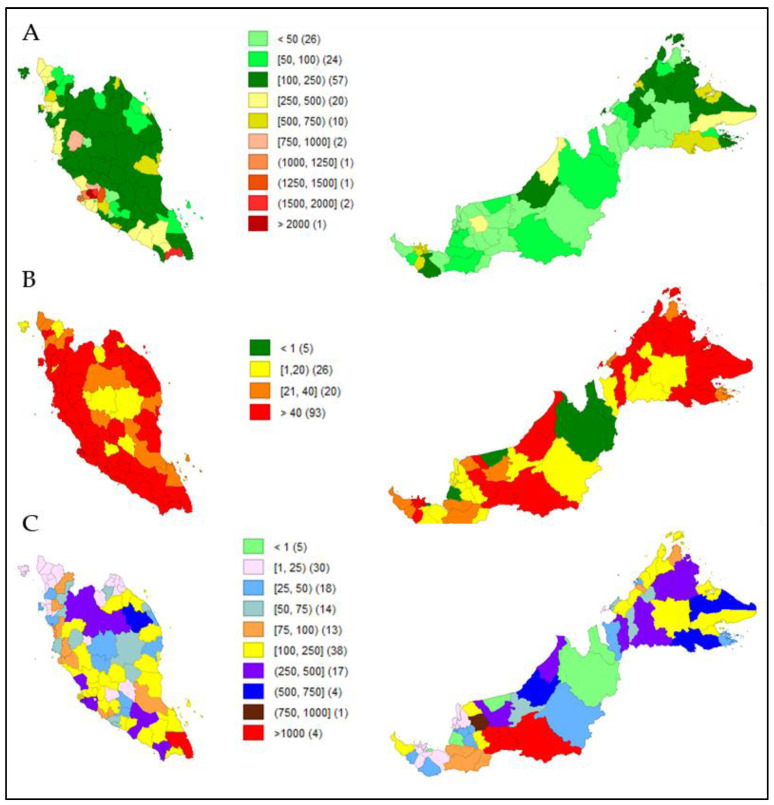
Choropleths showing district-wise heat distribution based on (**A**) total inhabitants per 1000 population (comparator); (**B**) total active cases (*n* = 51,476); (**C**) attack rate.

Figure 2 shows a panel series of choropleths. Figure 2B,C shows heat map visualizations based on district-wise active case counts and attack rates in relation to the comparator, Figure 2A, which populates total inhabitants according to districts in Malaysia (parameter values for all 144 districts are available as Supplementary File Table S1). Hotspots of active cases were mostly concentrated across districts with more than 250,000 inhabitants (Figure 2B). However, attack rates were relatively lower in dense populations in areas with less than 250,000 inhabitants (Figure 2C).

**Figure 3:**
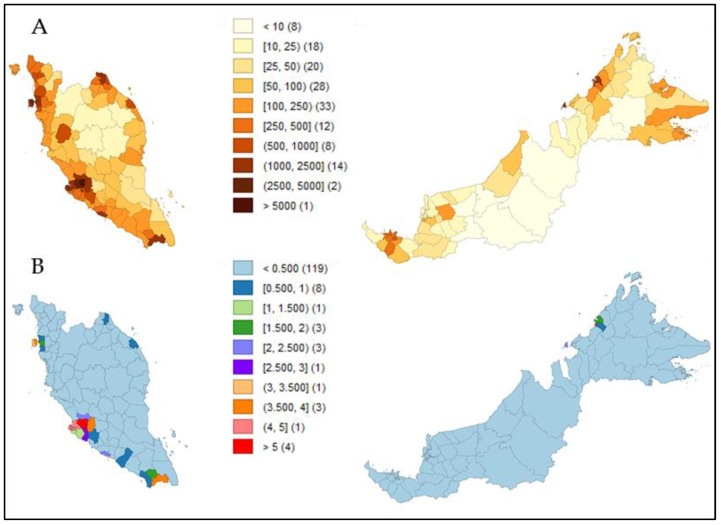
Choropleths showing district-wise heat distribution based on (**A**) population density (comparator); (**B**) infected people per km^2^.

We found that in districts with more than 250,000 inhabitants (Figure 2A) and with a density of more than 500 inhabitants/km^2^ (Figure 3A), approximately 1.5 people or more per km^2^ were infected with COVID-19 (Figure 3B).

### 3.2. Taxonomy of COVID-19 Cases and Population Density Based on District-Wise Hierarchical Cluster Analysis

**Figure 4:**
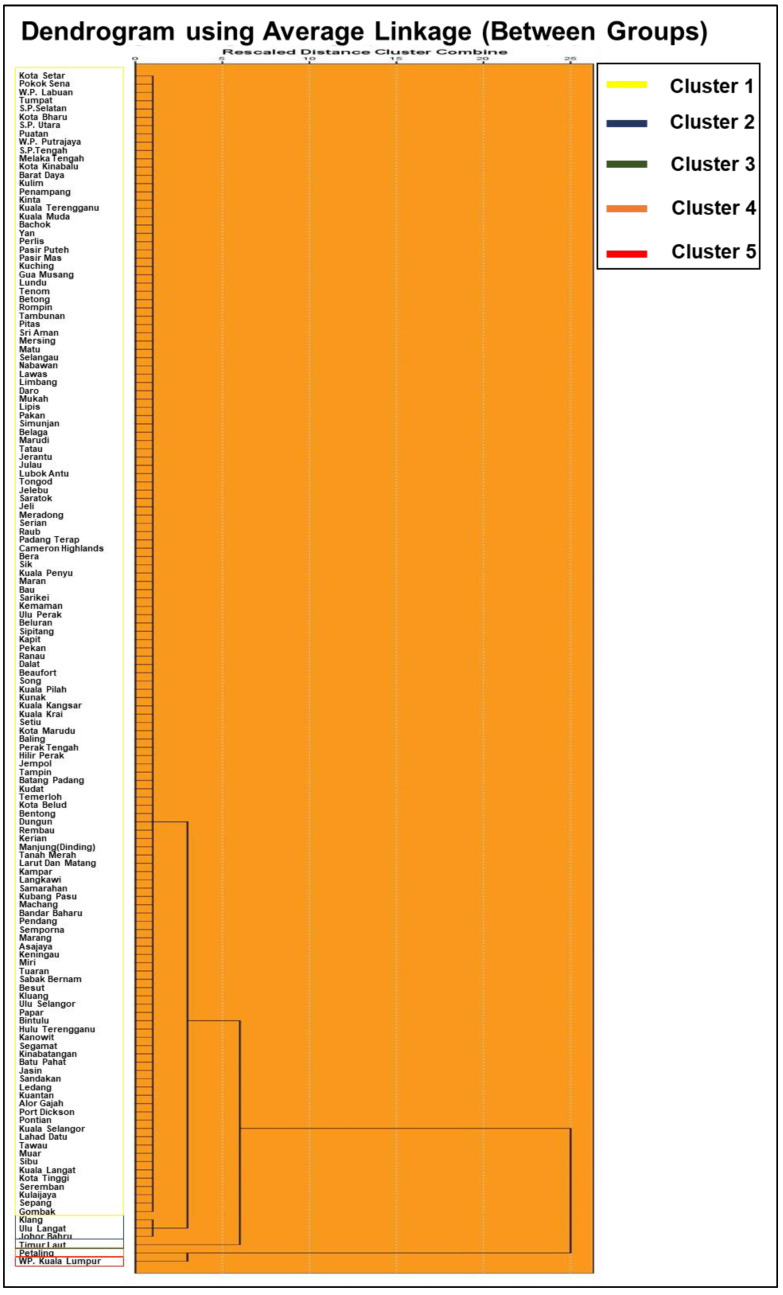
Dendrogram of cluster analysis that taxonomized COVID-19 cases and population density according to districts by average linkage method.

Figure 4 exhibits a dendrogram that clusters the districts according to active COVID-19 cases and population density for the entire study period. The average linkage method yielded five clusters. Cluster 1 comprises 138 districts with an average of 256 inhabitants per square kilometer with average cases accounting for 186 people. Cluster 2 is composed of 3 districts having an average of 1613 inhabitants per square kilometer, with average cases approximating 3136 people. Total inhabitants per square kilometer (number of cases) with average cases for clusters 3, 4, and 5 that comprised one district each (principally larger urbanized districts, cities, or metropolitan areas) accounted for 4893 (476), 4499 (7864), and 7812 (8014) people, respectively.

### 3.3. Correlations between Active COVID-19 Cases and Population Density

Table 1 shows correlations between active COVID-19 cases and population density at countrywide and regional levels. From the countrywide perspective, Malaysia showed a statistically significant positive correlation between active COVID-19 cases and population density (*r* = 0.784; 95% CI 0.781, 0.787; *p* < 0.001). Regional-based correlations found that the Central region had the strongest statistically significant positive relationship between active COVID-19 cases and population density (r = 0.912; 95% CI 0.911, 0.913; *p* < 0.001), followed by the Southern region (*r* = 0.731; 95% CI 0.728, 0.734; *p* < 0.001), the Northern region (*r* = 0.691; 95% CI 0.687, 0.695; *p* < 0.001), and the East Coast region (*r* = 0.501; 95% CI 0.496, 0.506; *p* = 0.007). Although it was statistically significant, East Malaysia had the weakest correlation between active COVID-19 cases and population density (*r* = 0.396; 95% CI 0.390, 0.402; *p* = 0.002). 

### 3.4. Propagation of Active COVID-19 Cases Influenced by Population Density

**Figure 5:**
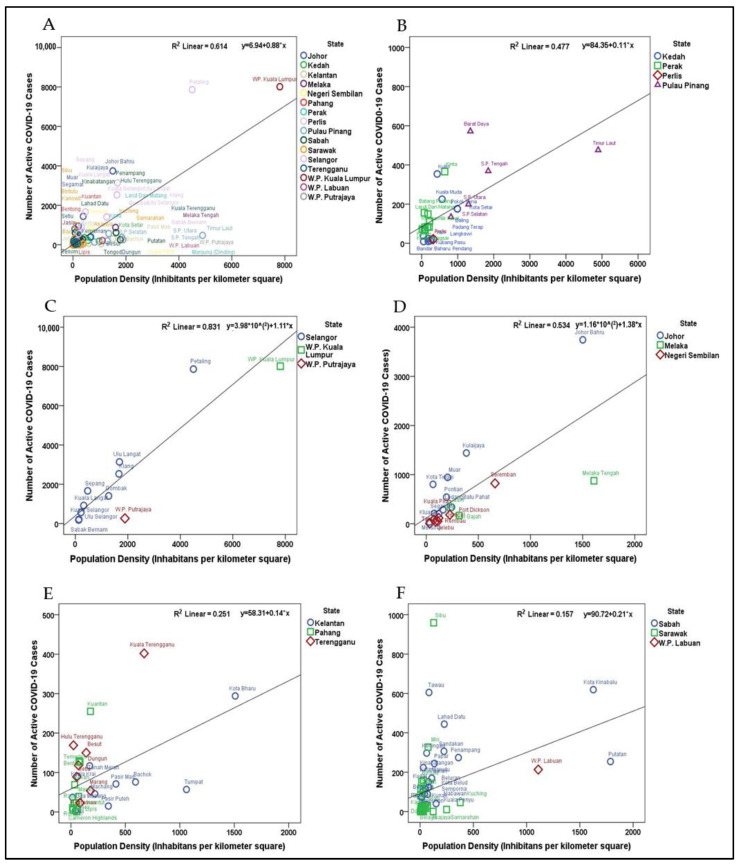
Point cloud population density and COVID-19 cases with model fitted simple regression lines. (**A**) Malaysia *R*^2^ = 0.614; (**B**) Northern region *R*^2^ = 0.477; (**C**) Central region *R*^2^ = 0.831; (**D**) Southern region *R*^2^ = 0.534; (**E**) East Coast region *R*^2^ = 0.251; (**F**) East Malaysia *R*^2^ = 0.157.

The influence of population density on the propagation of active COVID-19 cases in Malaysia is shown in Figure 5. The proportion of the variation that covers the spread of COVID-19, attributed directly to population density for each region in Malaysia, was interpreted through the coefficient of determination (*R*^2^) values. By using simple linear regression equations, *Y* = *B* + a*X*, the current study deduced the expected rise of COVID-19 cases as per the increase for one inhabitant/km^2^ in each region; in other words, the propagation effect yielding the number of active COVID-19 cases for each unit of population density at the given time point. Population density correlated with the spread of COVID-19 at the rate of 61.4% for the whole country. Notably, the countrywide point cloud revealed a propagation effect at the significance level of 5%, as each time the population density increased by 1 individual/km^2^, there was an increase in the number of active COVID-19 cases estimated at 0.88 for all districts in Malaysia (Figure 5A). 

For region-wise estimates, the rate at which population density explained the spread of COVID-19 for the Northern region was 47.7%. This region-wise point cloud estimation had a propagation effect at the significance level of 5%, as each time the population density increased by 1 individual/km^2^, there was an increase in the number of active COVID-19 cases estimated at 0.11 for all districts within the Northern region (Figure 5B). The rate at which population density explained the spread of COVID-19 for the Central region was the highest, at 83.1%. This region-wise point cloud estimation had a propagation effect at a significance level of 5%, as each time the population density increased by 1 individual/km^2^, there was an increase in the number of active COVID-19 cases estimated at 1.11 for all districts within the Central region (Figure 5C). Similarly, population density explained the spread of COVID-19 for the Southern region at a rate of 53.4%. This region-wise point cloud estimation had a propagation effect at the significance level of 5%, as each time the population density increased by 1 individual/km^2^, there was an increase in the number of active COVID-19 cases estimated at 1.38 for all districts within the Southern region (Figure 5D). 

Regarding the East Coast region, population density explained the spread of COVID-19 for this region at the rate of 25.1%. This region-wise point cloud estimation had a propagation effect at the significance level of 5%, as each time the population density increased by 1 individual/km^2^, there was an increase in the number of active COVID-19 cases estimated at 0.14 for all districts within the East Coast region (Figure 5E). Population density explained the spread of COVID-19 for East Malaysia at the lowest rate, 15.7%. This region-wise point cloud estimation had a propagation effect at the significance level of 5%, as each time the population density increased by 1 individual/km^2^, there was an increase in the number of active COVID-19 cases estimated at 0.21 for all districts within this region (Figure 5F). 

## 4. Discussion

There were two waves of COVID-19 cases in Malaysia between January and June 2020. The current third wave started in September 2020 and the surge in the number of cases is significant. While the previous two waves reported incidence cases in a logarithmic pattern, the current exponential growth of cases in Malaysia is somewhat difficult to decipher. Despite successful efforts to contain the outbreak in the previous two waves [14,32], the unexpected exponential rise of cases in the latter, third wave of contagion in Malaysia currently seems difficult to control with nonpharmacological interventions. Revisiting empirical observations from the classical Kermack–McKendrick model that postulates that the size of an epidemic is associated with dense populations [33], and coupling this with newer catalytic real-life meteorological, mobility or human interactions that can influence the spread and decay durations of epidemics, these factors could plausibly explain why widespread transmissions occur at a later time, as in Malaysia [12,17].

The point of propagation could have been initiated by the Sabah state elections on 26 September 2020, a potential mass gathering in a single state that spilled over into a national-level outbreak [34]. Limited testing and undetected asymptomatic cases could be a point of initiation for the geometric spread of infections when heterogenous mixing occurs via human mobility or migrations initiated from populations returning from mass gatherings [14,20]. This could plausibly explain why the index region (East Malaysia) had a relatively low correlation coefficient with COVID-19 cases as compared to other regions in Malaysia, as epidemic growths are principally influenced by the contact rates of people [18], coherently being facilitated by super-connected incubators such as the compactness of people or urban sprawl across megaregions or megacities within a country [35].

From the distribution-based choropleths and taxonomic synthesis, the current study observed active case counts to be widely distributed in larger districts, big cities, or metropolitan areas with a higher number of inhabitants or population density. Complementing distribution-wise observations, this study found a strong positive correlation between population density and the incidence of COVID-19 cases in the Central region of Malaysia—a region with a highly dense population composed of urbanized districts, cities, or metropolitan areas within the state of Selangor and Kuala Lumpur city. These findings are consistent with recent investigations from Algeria [13], Bangladesh [16], and India [11].

Although population density explained the highest spread of COVID-19 cases for the Central region, the propagation effect juxtaposed between two regions, yielding an approximate 27% higher risk of infections for districts within the Southern region, even though population density was much lower when compared to the Central region. Such observations could be attributed to proxy drivers of contact rates, contributing by active trade or business activities within the city of Johor Bahru, coupled with high human mobility to a neighboring country, thus escalating the rate of a susceptible proportion of people to infections.

The identification of hotspots reflects an attack rate threshold, and it incorporates population density rather than a straight incidence-rate value. A higher incidence rate is bound to exist in high-population areas as compared to low-density areas based on the availability of people susceptible to infections; however, the current study noted that the attack rate of the COVID-19 epidemic was higher in smaller districts than in larger ones (Figure 2C). This finding was consistent with a previous study from China [36]. Such observations should be interpreted with caution, as attack rates of the epidemic could possibly be attributed to population size, in which districts or cities with smaller number of inhabitants will have higher attack rates as compared to others, as attack rate serves as a relative metric for interpretation. This could support the notion advocated by previous researchers when the size of the population outweighs population density in the spread of COVID-19 [16,37]. Another plausibility of such inconsistency could be attributed to the cut-off standard set by Malaysia to classify areas and districts with more than 40 incidence cases as potential red zones or hotspots [38,39]. The reason for this move was to relax the stringent containment measures that were implemented for the whole country to continue, and to subsequently shift toward a more targeted approach, meaning districts or areas with more than 40 reported active cases would be placed under enhanced containment measures with rigorous contact tracings, while lower incidence cases would practice somewhat relaxed standard operating procedures to allow economic and social activities, yet facilitate people’s movement to a certain threshold. At the time of this study, a national lockdown was not implemented, and interventions were solely based on the targeted implementation of a movement control order (MCO) and standard operating procedures. Incidence count will not equal an attack rate, and attack rates principally rely on potential exposure (contact with an infected person). When levels of interventions are different, people’s communication and mobility vary, and so does transmissibility, regardless of density.

The seriousness of infectiousness (the extent to which COVID-19 has affected the population) across districts could be influenced by the mobile capacity of inhabitants, migrations to other cities or districts, health infrastructure availability, transportation, and high economic activities or densely occupied migrant workers [36]. Such postulations could possibly be explained in the context of the Bukit Mambong district integrated within the Kapit division of Sarawak, which showed high epidemic seriousness from the distributional heat map. The division is a vibrant commercial, social, and tourism center that attracts visitors and promotes interactions among people alongside longhouse communities and timber camp migrants, thus increasing the probability of infections. From the Peninsular perspective, the Kota Tinggi district within the state of Johor has been a tourist attraction with good transportation infrastructure and railway lines, and highway accessibilities linking to East Coast cities and Kuala Lumpur, thus becoming a potential catalyst to receive people from high COVID-19 epidemic areas.

The current study could have important public health implications. This study confirmed that population density had direct correlations with COVID-19 infections, yet was unable to demonstrate the magnitude of the effect on how much the risk of infection could be attributed to people when a unit of density is increased in a particular district. Such established risks, weighted geographically, could direct the crafting of standard operating procedures and the implementation of containment measures based solely on “place”, rather than implementing a generalized measure of multipronged interventions which waste financial and healthcare resources.

The limitations of this study should be acknowledged. The ecological correlation design employed in this study is subject to ecological fallacy. Although the current study potentially demonstrated possible correlations between population density and COVID-19 incidence cases at the population aggregate level, the findings cannot be interpolated to an individual level. The spatial analysis clearly demonstrated the distribution of COVID-19 cases across districts in Malaysia, but the spatial-temporal relationships with potential predictors or confounders such as transmission clusters in workplace or factory workers, industries, nursing homes, prisons, places with poor ventilation, religious places, and cultural activities such as festivals or celebrations were not tested or explored. The sampling time frame was relatively short and did not capture the impact of the new vaccination policy that was implemented in late February 2021, hence causing the current study to not be sufficiently powered to explore future projections of interacted correlations between the variables of suppression strategies, population density, and infections.

## 5. Conclusions

Population density is a factor in the spread of COVID-19 cases in Malaysia. It would be appropriate to draft standard operating procedures that consider population density as a risk factor for COVID-19 spread and weigh them geographically prior to executing nontherapeutic interventions in the quest to control the epidemic.

## Figures and Tables

**Table 1:** Correlation between active COVID-19 cases and population density.

Region	*r* (95% CI)	*p*-Value
Malaysia	0.784 (0.781, 0.787)	<0.001
Northern region	0.691 (0.687, 0.695)	<0.001
Central region	0.912 (0.911, 0.913)	<0.001
Southern region	0.731 (0.728, 0.734)	<0.001
East Coast region	0.501 (0.496, 0.506)	0.007
East Malaysia	0.396 (0.390, 0.402)	0.002

## Data Availability

Publicly available datasets were utilized in this study. These data can be found here: https://covid-19.moh.gov.my/ (accessed on 4 February 2021); https://www.dosm.gov.my/v1/ (accessed on 4 February 2021); https://www.jupem.gov.my/ (accessed on 4 February 2021).

## Supplementary Materials

The following are available online at https://www.mdpi.com/article/10.3390/ijerph18189866/s1, Table S1: District-Wise Population Level Information and COVID-19 Cases.
