# Supplementary material for: Influence of Population Density for COVID-19 Spread in Malaysia: An Ecological Study
Source: Int J Environ Res Public Health. 2021 Sep 18;18(18):9866. doi: 10.3390/ijerph18189866 (PMC8468130; doi:10.3390/ijerph18189866)
Supplement: Supplementary file 1 [file ijerph-18-09866-s001.zip › ijerph-1365748-supplementary.pdf]

Table S1: District-Wise Population Level Information and COVID-19 Cases

No	State	District	Total population (x1000)	Area in square kilometre	Population density	Cases as on 4/2/2021
1	Johor	Batu Pahat	482.2	1966.31	245.2309	338
2		Johor Bahru	1600.3	1065.84	1501.445	3743
3		Kluang	347.1	2864.53	121.1717	202
4		Kota Tinggi	227.7	3488.56	65.27048	802
5		Kulaijaya	291	755.51	385.1703	1440
6		Ledang	157.7	976.76	161.4521	285
7		Mersing	83.8	2856.59	29.33568	37
8		Muar	285.3	1392.58	204.8715	944
9		Pontian	180.5	932.64	193.5366	542
10		Segamat	218.7	2866.56	76.29354	211
11	Kedah	Baling	156.4	1528	102.356	67
12		Bandar Baharu	48.6	269	180.6691	10
13		Kota Setar	417.8	422	990.0474	176
14		Kuala Muda	521.2	922	565.2928	225
15		Kubang Pasu	254.2	948	268.1435	21
16		Kulim	329.8	767	429.987	354
17		Langkawi	111.5	466	239.2704	13
18		Padang Terap	73	1357	53.79514	9
19		Pendang	109.9	627	175.2791	24
20		Pokok Sena	417.8	422	990.0474	176
21		Sik	78.2	1634	47.85802	37
22		Yan	78.8	241	326.971	21
23	Kelantan	Bachok	165.8	279.51	593.1809	76
24		Gua Musang	116.3	8214.3	14.15824	37
25		Jeli	51.9	1330.48	39.00848	3
26		Kota Bharu	608.6	403.26	1509.2	294
27		Kuala Krai	137.9	2287.1	60.2947	90
28		Machang	115.9	528.51	219.2958	46
29		Pasir Mas	236.4	572.38	413.0123	71
30		Pasir Puteh	146	424.94	343.5779	15
31		Tanah Merah	152.1	884.14	172.0316	117
32		Tumpat	191	180	1061.111	57
33	Melaka	Alor Gajah	212.1	674	314.6884	169
34		Jasin	156.6	679	230.6333	334
35		Melaka Tengah	579	360	1608.333	875
36	Negeri Sembilan	Jelebu	45.2	1349.9	33.48396	5
37		Jempol	132	1480.9	89.13499	44
38		Kuala Pilah	74.5	1039.16	71.69252	84
39		Port Dickson	130.5	575.76	226.6569	184
40		Rembau	49	406.2	120.6302	117

41		Seremban	625.2	947.82	659.6189	822
42		Tampin	95.5	857.35	111.3897	51
43	Pahang	Bentong	135.3	1831	73.89405	129
44		Bera	112.2	2228	50.35907	1
45		Cameron Highlands	43.7	712	61.3764	6
46		Jerantut	105.3	7561	13.92673	10
47		Kuantan	529.5	2960	178.8851	255
48		Lipis	103.9	5198	19.98846	5
49		Maran	134.5	1903	70.67788	39
50		Pekan	129.3	3805	33.9816	69
51		Raub	107.8	2269	47.50992	23
52		Rompin	135	5247	25.72899	23
53		Temerloh	189.9	2251	84.36251	126
54	Perak	Batang Padang	130.5	1794	72.74247	156
55		Hilir Perak	156.3	1728	90.45139	82
56		Kampar	108.6	669.8	162.138	85
57		Kerian	197.6	958	206.263	83
58		Kinta	835.8	1305	640.4598	366
59		Kuala Kangsar	176.3	2541	69.38213	72
60		Larut Dan Matang	365	2105	173.3967	149
61		Manjung (Dinding)	256.4	1171	218.9582	112
62		Perak Tengah	113.2	1279	88.50665	67
63		Ulu Perak	104	6563	15.84641	72
64	Perlis	Perlis	262	819	319.9023	21
65	Pulau Pinang	Barat Daya	234.1	173.45	1349.668	572
66		S.P. Tengah	433.4	234.89	1845.119	369
67		S.P. Utara	340.6	262.52	1297.425	199
68		S.P. Selatan	195.8	241	812.4481	134
69		Timur Laut	583.2	119.18	4893.438	476
70	Sabah	Beaufort	83.4	1742	47.876	116
71		Beluran	133.5	8345	15.9976	72
72		Keningau	219.1	3546.4	61.78096	297
73		Kinabatangan	198.1	6630	29.87934	223
74		Kota Belud	111.8	1391	80.37383	124
75		Kota Kinabalu	572.6	352.1	1626.243	619
76		Kota Marudu	81.3	1924	42.25572	89
77		Kuala Penyu	24.5	455	53.84615	36
78		Kudat	101.7	1292.2	78.70299	151
79		Kunak	80.3	1134	70.81129	88
80		Lahad Datu	259.2	1138.8	227.608	444
81		Nabawan	32	6089	5.255379	17
82		Papar	167.3	1248	134.0545	244

83		Penampang	152.9	424.72	360.0019	274
84		Pitas	45.4	1419	31.99436	31
85		Putatan	71.5	40	1787.5	254
86		Ranau	114	3622	31.47432	104
87		Sandakan	510.6	2275	224.4396	306
88		Semporna	172.9	1149	150.4787	40
89		Sipitang	45.1	2743	16.44185	75
90		Tambunan	43.4	1352	32.10059	18
91		Tawau	514	6149	83.59083	605
92		Tenom	68.8	2418	28.45327	19
93		Tongod	44.2	10092	4.379707	10
94		Tuaran	128.2	1170	109.5726	169
95	Sarawak	Asajaya	37.3	302.8	123.1836	0
96		Bau	61.4	884.4	69.4256	29
97		Belaga	43.9	19403.19	2.262515	1
98		Betong	72.4	2493.89	29.03095	22
99		Bintulu	226.3	7220.36	31.34193	159
100		Dalat	22.9	905.29	25.29576	113
101		Daro	37.2	1956.27	19.01578	1
102		Julau	18.4	1703.39	10.80199	12
103		Kanowit	33.8	2253.54	14.99862	151
104		Kapit	64.8	15595.6	4.155018	80
105		Kuching	702.7	1862.81	377.2258	46
106		Lawas	45.5	3811.91	11.93627	3
107		Limbang	56.1	3978.1	14.10221	0
108		Lubok Antu	32.7	3142.55	10.40556	8
109		Lundu	38.7	1812.34	21.35361	31
110		Marudi	75.7	22069.96	3.430002	0
111		Matu	21	1600.05	13.12459	25
112		Meradong	34.3	719	47.70515	16
113		Miri	352.3	4707.11	74.84423	327
114		Mukah	51.5	2536	20.30757	0
115		Pakan	18.3	924.6	19.79234	5
116		Samarahan	101.3	407.08	248.8454	10
117		Saratok	53.6	1586.9	33.77655	0
118		Sarikei	66.5	985	67.51269	18
119		Selangau	27	3795	7.114625	23
120		Serian	104.4	2039.89	51.17923	18
121		Sibu	284.7	2229.75	127.6825	959
122		Simunjan	46.2	2217.67	20.83268	3
123		Song	24.1	3935.19	6.124228	117
124		Sri Aman	77.2	2323.7	33.22288	28
125		Tatau	36.5	4945.85	7.379925	4
126	Selangor	Gombak	828.8	652.7	1269.802	1406

127		Klang	1040.9	626.8	1660.657	2529
128		Kuala Langat	274.7	857.7	320.2752	918
129		Kuala Selangor	255.6	1178.4	216.9043	566
130		Petaling	2190.7	486.9	4499.281	7864
131		Sabak Bernam	128.3	997.1	128.6732	178
132		Sepang	261.4	556.1	470.0593	1665
133		Ulu Langat	1392.1	828.8	1679.657	3137
134		Ulu Selangor	241.8	1745.7	138.5118	216
135	Terengganu	Besut	172.5	1234	139.7893	150
136		Dungun	189.6	2735	69.32358	118
137		Hulu Terengganu	89	3875	22.96774	169
138		Kemaman	212.1	2536	83.63565	23
139		Kuala Terengganu	418.5	622.7	672.0732	402
140		Marang	119.4	667	179.0105	54
141		Setiu	68.6	1304	52.60736	89
142	W.P. Kuala Lumpur	W.P. Kuala Lumpur	1895.7	242.7	7810.878	8014
143	W.P. Labuan	W.P. Labuan	101.7	91.64	1109.777	213
144	W.P. Putrajaya	W.P. Putrajaya	93.1	49.3	1888.438	262
